# Commercial AI solutions in detecting COVID-19 pneumonia in chest CT: not yet ready for clinical implementation?

**DOI:** 10.1007/s00330-021-08409-4

**Published:** 2021-12-23

**Authors:** Florian Jungmann, Lukas Müller, Felix Hahn, Maximilian Weustenfeld, Ann-Kathrin Dapper, Aline Mähringer-Kunz, Dirk Graafen, Christoph Düber, Darius Schafigh, Daniel Pinto dos Santos, Peter Mildenberger, Roman Kloeckner

**Affiliations:** 1 Department of Diagnostic and Interventional Radiology, University Medical Center of the Johannes Gutenberg University Mainz, Langenbeckst. 1, 55131 Mainz, Germany; 2 Department of Neuroradiology, University Medical Center of the Johannes Gutenberg University Mainz, Mainz, Germany; 3 Department of Radiology, University Hospital of Cologne, Cologne, Germany

**Keywords:** Radiology, COVID-19, Pneumonia, Computed tomography, Artificial intelligence

## Abstract

**Objectives:**

In response to the COVID-19 pandemic, many researchers have developed artificial intelligence (AI) tools to differentiate COVID-19 pneumonia from other conditions in chest CT. However, in many cases, performance has not been clinically validated. The aim of this study was to evaluate the performance of commercial AI solutions in differentiating COVID-19 pneumonia from other lung conditions.

**Methods:**

Four commercial AI solutions were evaluated on a dual-center clinical dataset consisting of 500 CT studies; COVID-19 pneumonia was microbiologically proven in 50 of these. Sensitivity, specificity, positive and negative predictive values, and AUC were calculated. In a subgroup analysis, the performance of the AI solutions in differentiating COVID-19 pneumonia from other conditions was evaluated in CT studies with ground-glass opacities (GGOs).

**Results:**

Sensitivity and specificity ranges were 62–96% and 31–80%, respectively. Negative and positive predictive values ranged between 82–99% and 19–25%, respectively. AUC was in the range 0.54–0.79. In CT studies with GGO, sensitivity remained unchanged. However, specificity was lower, and ranged between 15 and 53%. AUC for studies with GGO was in the range 0.54–0.69.

**Conclusions:**

This study highlights the variable specificity and low positive predictive value of AI solutions in diagnosing COVID-19 pneumonia in chest CT. However, one solution yielded acceptable values for sensitivity. Thus, with further improvement, commercial AI solutions currently under development have the potential to be integrated as alert tools in clinical routine workflow. Randomized trials are needed to assess the true benefits and also potential harms of the use of AI in image analysis.

**Key Points:**

*• Commercial AI solutions achieved a sensitivity and specificity ranging from 62 to 96% and from 31 to 80%, respectively, in identifying patients suspicious for COVID-19 in a clinical dataset*.

*• Sensitivity remained within the same range, while specificity was even lower in subgroup analysis of CT studies with ground-glass opacities, and interrater agreement between the commercial AI solutions was minimal to nonexistent*.

*• Thus, commercial AI solutions have the potential to be integrated as alert tools for the detection of patients with lung changes suspicious for COVID-19 pneumonia in a clinical routine workflow, if further improvement is made*.

**Supplementary Information:**

The online version contains supplementary material available at 10.1007/s00330-021-08409-4.

## Introduction

The coronavirus disease 2019 (COVID-19) was identified in 2019 in China [1]. Since then, it has spread over the world and become a heavy burden on health care systems. The identification of patients infected by the severe acute respiratory syndrome coronavirus 2 (SARS-CoV-2) is very important in controlling the spread of the disease. The diagnosis primarily relies on molecular biological testing using the real-time polymerase chain reaction (RT-PCR). Imaging is useful in the evaluation of patients with suspected or known COVID-19: for example, to rule out alternative diagnoses such as pulmonary embolism [2]. In a meta-analysis, the pooled sensitivity for chest CT in the diagnosis of COVID-19 was 94%, and the positive predictive value ranged from 1.5 to 30.7% [3]. However, the true sensitivity of chest CT is overestimated [4]. While the sensitivity was high (67–100%), the specificity was relatively low (25–80%). Therefore, the World Health Organization recommends that CT should not be used to screen for COVID-19 [5].

To harmonize and standardize report communication with the referring physicians, the COVID-19 Reporting and Data System (CO-RADS) classifies CT findings into five categories according to the probability of COVID-19 pneumonia [6]. Depending on the chosen threshold, the sensitivity and specificity for the diagnosis of COVID-19 vary considerably [2]. Interrater agreement in the differentiation of COVID-19 from other atypical pneumonias is only moderate and reader expertise does not correlate with accuracy [7].

Artificial intelligence (AI) promises great potential in image analysis, such as in evaluating chest X-rays and mammograms [8, 9]. Many deep-learning models pretend to accurately detect COVID-19 in chest CT and even to differentiate it from community-acquired pneumonia or other lung conditions. In four real-life datasets, sensitivity ranged from 0.81 to 0.95, and specificity ranged from 0.82 to 0.97 [10–13].

However, despite these encouraging first results, translation of such deep-learning models into clinical routine is currently not feasible, due to significant methodological weaknesses [14].

In this study, we aimed to evaluate the performance of several commercial AI solutions in the differentiation of COVID-19 pneumonia from other lung conditions in a dual-center clinical dataset.

## Methods

This study was approved by the responsible ethics committees (Ethics Committee of the Medical Association of Rhineland Palatinate, Mainz, permit number 2020–15,066, and Ethics Commission of Cologne University’s Faculty of Medicine, Cologne, Germany, permit number 20–1281). All CT studies were fully anonymized. STARD 2015 guidelines for the reporting in diagnostic accuracy studies were followed during the conduct of the study and in the drafting of this manuscript (Supplemental Fig. 1) [15].

### Dataset

We retrospectively compiled a clinical dataset of 500 CT studies of the chest with and without intravenous contrast administration from two different tertiary care institutions. CT scans were performed on a 256-, 64-, or 16-slice CT scanner (Philips).

**Fig. 1:**
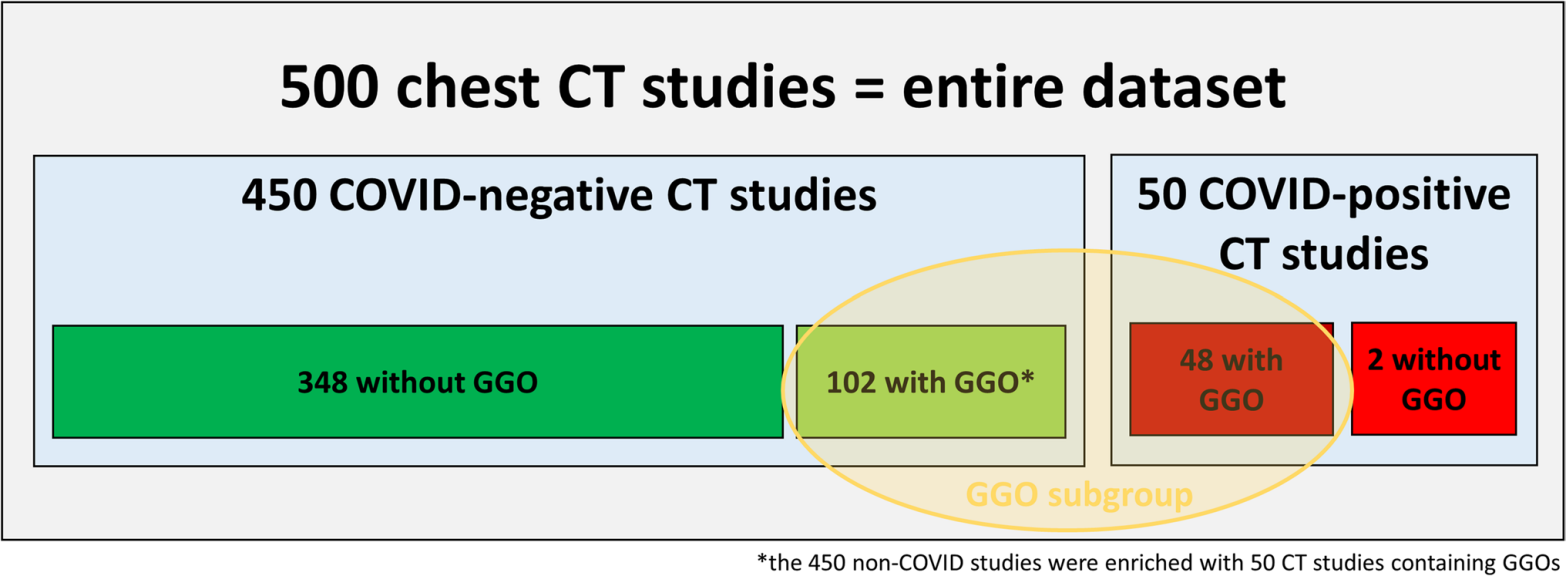
Composition of the dataset: COVID-19 status and presence of ground-glass opacities (GGOs)

The dataset consisted of 50 CT scans with RT-PCR–proven COVID-19 diagnoses and 450 COVID-19–negative CT scans comprising 400 CT scans randomly selected from all chest CT scans in 2018 and 50 scans from a predefined clinical dataset from 2018 with verified ground-glass opacities (GGOs) to ensure that a minimum of non-COVID-19 CT scans with GGOs was guaranteed (Fig. 1). All patients with COVID-19 presented in the acute stage and were either transferred from smaller hospitals or presented themselves to the emergency room. For all 500 CT scans, a radiologist with 3 years of experience in chest imaging categorized the pulmonary changes as pleural effusion, ground-glass opacities (GGOs), consolidation, tumor, or pulmonary venous congestion. Extensiveness was estimated by visual assessment of the GGOs and consolidations in comparison with the total lung volume. Form, predominantly horizontal and predominantly vertical distribution, lobe involvement, and density were assessed as previously reported [16, 17]. Table 1 illustrates the categorized findings for the whole dataset. Additional detailed information on the reasons for imaging in the 450 patients with other lung conditions can be found in Supplementary Table 2.

**Table 1:** Dataset characteristics

	Entire dataset	Proven SARS-CoV-2 infection	Other lung conditions (2018)
Number of CT studies	500	50	450
Sex (F:M)	193:307	22:28	171:279
Age (standard deviation)	61.90 (15.48)	58.64 (14.47)	62.26 (15.55)
Intravenous contrast administration, *n* (%)	322 (64.4)	8 (16.0)	314 (69.8)
No visible lung pathology, *n* (%)	213 (42.6)	2 (4.0)	211 (46.9)
Pleural effusion, *n* (%)	89 (17.8)	5 (10.0)	84 (18.7)
Ground-glass opacity (GGO)^a^, *n* (%)	150 (30.0)	48 (96.0)	102 (22.7)
Consolidation (%)	114 (22.8)	28 (56.0)	86 (19.1)
Mild	43	11	32
Moderate	40	13	27
Marked	31	4	27
Tumor, *n* (%)	55 (11.0)	1 (2.0)	54 (12.0)
Solitary nodule	31 (6.2)	1 (2.0)	30 (6.7)
Multiple nodules	24 (4.8)	0	24 (5.3)
Pulmonary venous congestion, *n* (%)	67 (13.4)	1 (2.0)	66 (14.7)

^a^Additional information on the morphologic characteristics and causes of the GGOs can be found in Supplementary Table 2

To better characterize our study dataset, all 50 CT scans of patients with proven SARS-CoV-2 infection were categorized according to CO-RADS [6] by three readers in consensus. The residents had, respectively, 2 and 3 years of training in thoracic imaging and lung CT. The board-certified radiologist had 10 years’ experience in thoracic imaging and lung CT, including the imaging of infectious diseases of the lung. The raters had no information on the course of disease and the clinical outcomes of the patients. Consensus reading was performed in those cases in which the three raters did not agree (17 scans). In 38 CT scans, CO-RADS 5 was assigned, whereas 8 CT scans were categorized as CO-RADS 4. Two CT scans were respectively classified as CO-RADS 1 and CO-RADS 3. A total of 20 (40.0%) patients with a PCR-proven SARS-CoV-2 infection had only GGOs, 28 (56.0%) patients showed GGOs in combination with other imaging signs of COVID-19, and 2 (4.0%) SARS-CoV-2–positive patients had no signs of a SARS-CoV-2 infection. Among the 50 patients with PCR-proven SARS-CoV-2 infection, 16 (32.0%) had a bacterial superinfection.

### AI solutions

From June 2020 to February 2021, we contacted nine companies and offered to evaluate their AI solutions for COVID-19 detection on our independent dual-center dataset containing 500 anonymized chest CT scans. At this time, five companies did not have a ready-to-use tool for differentiating between COVID-19 pneumonia and other lung conditions, or we were unable to sign a data-transfer agreement acceptable to both parties. Four companies finally agreed to participate in our challenge and signed the data-transfer agreement: contextflow (contexflow DEMO Lung CT version 1.1.8), icometrix (Icolung version 0.6.0), Infervision (InferRead CT Pneumonia version 1), and Siemens (Pneumonia Assessment version 2.0). At the time of the initial evaluation of our chest CT collection, all solutions were under development and not purchasable. Thus, we had no information on the composition of the training set used for the development of the algorithm and the initial performance of the algorithm. More information regarding the evaluated algorithms has become publicly available, and we have contacted the companies explicitly for more detailed information on the versions used for the evaluation of our dataset (Supplementary Table 3). The proportion of COVID-19 cases among the 500 CT scans was not disclosed to the companies. The task was to categorize each study as “COVID-19 pneumonia suspect” or “not COVID-19 pneumonia suspect,” which was the primary endpoint of this study. Thus, one requirement for participating in this study was that the company was offering a tool for the detection of COVID-19 pneumonia in a dataset of chest CT (Supplementary Table 3). The four companies were able to download the lung window in DICOM format of the anonymized CT studies to start their analysis. All image processing and analysis was performed by the companies themselves. The result was an Excel file with the binary annotations “COVID-suspected” and “other lung condition.” As we sent the companies the raw DICOM files, we had no information about the segmentation accuracies, volume calculations, or probability scores of the algorithms. Three companies were able to process CT studies with and without intravenous (i.v.) contrast administration, whereas one company focused on CT studies without i.v. contrast. To compare the results of the AI solutions with those of a radiologist, we had all the images rated by two board-certified radiologists with, respectively, 7 and 10 years’ experience. Both were blinded to the incidence of COVID-19 cases in our dataset and had, similar to the companies, no clinical information. Results are shown without attachment to a company name, due to data safety reasons and the lack of an agreement.

### Statistical analysis

Statistical analyses were performed using R (A Language and Environment for Statistical Computing, R Foundation for Statistical Computing, http://www.R-project.org). We compared the results of the companies with the gold standard (RT-PCR–proven SARS-CoV-2 infection) and calculated the numbers of true positives, true negatives, false positives, and false negatives.

With these numbers, we calculated the sensitivity, specificity, positive predictive value (PPV), and negative predictive value (NPV). Furthermore, the area under the curve (AUC) was calculated for each AI solution. For all analyses, we used CT studies classified as CO-RADS ≥ 3 to ensure that the included scans showed pertinent changes.

In subgroup analyses, the sensitivity, specificity, PPV, NPV, and AUC in CT studies with GGO were calculated for differentiating COVID-19 pneumonia from other lung conditions. Agreement between the four AI solutions was measured using Cohen’s kappa coefficient (*k*).

## Results

Two AI solutions successfully processed 498 CT studies, whereas the other solutions processed 497 and 174 studies. Table 2 demonstrates the performance measurements of the AI solutions in differentiating COVID-19 pneumonia from other lung conditions.

**Table 2:** Performance of the tools in differentiating COVID-19 pneumonia (CO-RADS ≥ 3) versus from lung conditions

	Company 1	Company 2	Company 3	Company 4	Radiologist 1	Radiologist 2
Studies analyzed	497	174^a^	498	498	498	498
TP	46	30	30	41	44	38
TN	278	42	360	270	394	412
FP	171	93	90	180	56	38
FN	2	9	18	7	4	10
Sensitivity	0.96	0.77	0.62	0.85	0.92	0.79
Specificity	0.62	0.31	0.80	0.60	0.88	0.92
PPV	0.21	0.24	0.25	0.19	0.44	0.5
NPV	0.99	0.82	0.95	0.97	0.99	0.98
AUC	0.79	0.54	0.71	0.73	0.90	0.85

^a^This AI tool processed only CT studies without i.v. contrast administration. *CO-RADS*, COVID-19 Reporting and Data System; *TP*, true positives; *TN*, true negatives; *FP*, false positives; *FN*, false negatives; *PPV*, positive predictive value; *NPV*, negative predictive value; *AUC*, area under the curve

The sensitivity and specificity ranges were 62–96% and 31–80%, respectively. The negative predictive value and positive predictive value ranges were 82–99% and 19–25%, respectively. The AUC for studies with CO-RADS ≥ 3 ranged from 0.54 to 0.79 (Supplemental Fig. 2). In comparison, the radiologists’ estimations of sensitivity and specificity were in the ranges 79–92% and 88–92%, respectively (Table 2).

In a subgroup analysis, the performance of the four AI solutions was measured for CT studies with GGO (Table 3). The subgroup of 150 CT scans with GGOs was built as shown in Fig. 1. Detailed information on the morphologic and clinical characteristics of the GGO subgroup can be found in Supplementary Table 1. In those cases, the sensitivity did not change but the specificity dropped to a range of 15–53%. The negative predictive value was also lower than in all cases, in the range 72–89%. The AUC for studies with GGO was in the range 0.54–0.69 (Supplemental Fig. 3). In comparison, the radiologists’ estimations of sensitivity and specificity were in the ranges 79–92% and 52–62%, respectively, for the subgroup of patients with GGOs (Table 3).

**Table 3:** Subgroup analysis. Performance of the tools in differentiating between COVID-19 lung infections (CO-RADS ≥ 3) and other lung conditions in CT studies with GGO. This subgroup consisted of 48 cases with proven SARS-CoV-2 infection and the presence of GGOs, and 102 cases with other lung conditions and the presence of GGOs

	Company 1	Company 2	Company 3	Company 4	Radiologist 1	Radiologist 2
Studies analyzed	149	88^a^	150	150	150	150
TP	46	30	30	41	44	38
TN	15	19	47	54	53	68
FP	86	30	55	48	49	34
FN	2	9	18	7	4	10
Sensitivity	0.96	0.77	0.62	0.85	0.92	0.79
Specificity	0.15	0.39	0.46	0.53	0.52	0.67
PPV	0.35	0.50	0.35	0.46	0.47	0.53
NPV	0.88	0.68	0.72	0.89	0.93	0.87
AUC	0.55	0.58	0.54	0.69	0.72	0.73

^a^This AI tool processed only CT studies without i.v. contrast administration. *CO-RADS*, COVID-19 Reporting and Data System; *GGO*, ground-glass opacity; *TP*, true positives; *TN*, true negatives; *FP*, false positives; *FN*, false negatives; *PPV*, positive predictive value; *NPV*, negative predictive value; *AUC*, area under the curve

Interrater agreement between company 1 and company 3 (0.37) as well as between company 1 and company 4 (0.28) was minimal, and no interrater agreement at all could be found in all other constellations (Table 4).

**Table 4:** Subgroup analysis. Performance of the tools in differentiating between COVID-19 pneumonia (CO-RADS ≥ 3) and other lung conditions in CT studies with GGO

	Company 1	Company 2	Company 3	Company 4
Company 1	/	− 0.03	0.37	0.28
Company 2	− 0.03	/	− 0.14	0.09
Company 3	0.37	− 0.14	/	0.08
Company 4	0.28	0.09	0.08	/

0–0.20, none; 0.21–0.39, minimal; 0.40–0.59, weak; 0.60–0.79, moderate; 0.80–0.90, strong; above 0.90, almost perfect

**Fig. 2:**
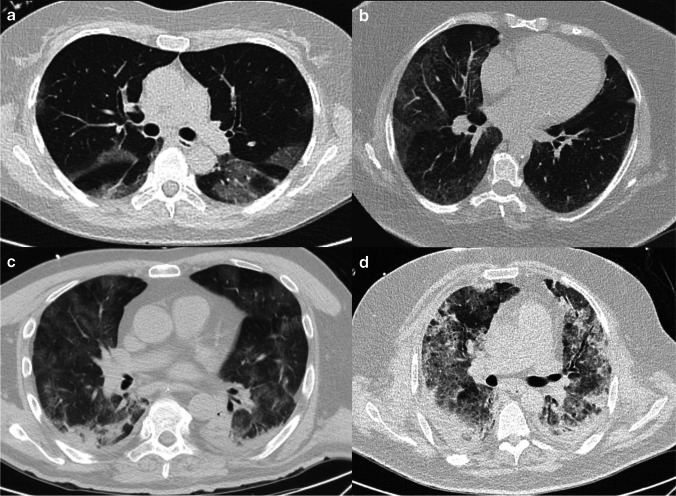
**a**–**d** Examples of four different patients with RT-PCR–proven SARS-CoV-2 infection, all of which were correctly categorized as COVID-19 suspect

**Fig. 3:**
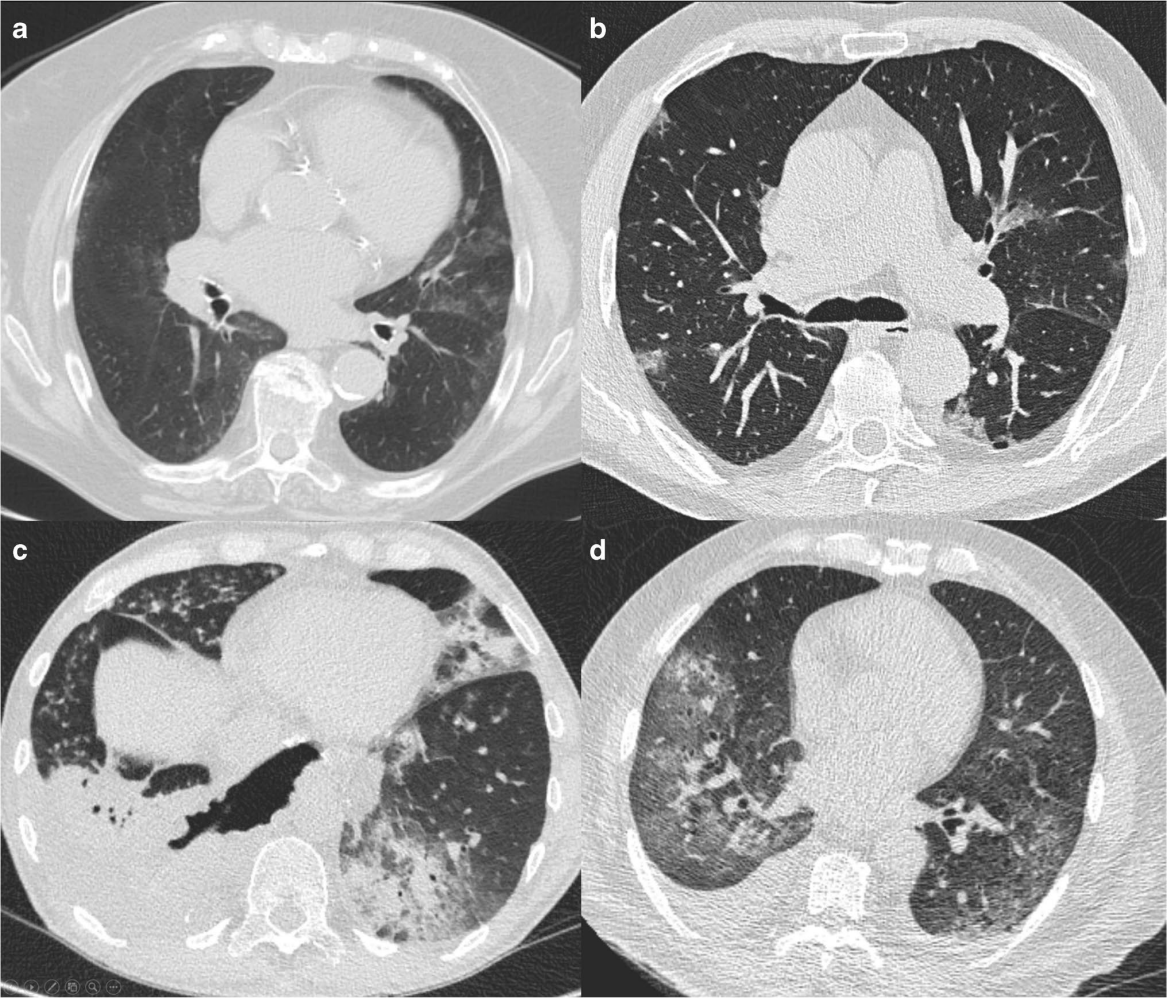
**a**–**d** Examples in which all AI solutions categorized the CT studies from 2018 as COVID-19 suspect

Figure 2 and Fig. 3 illustrate examples of CT studies used in our dataset.

## Discussion

In this study, we challenged four AI solutions developed to identify COVID-19 pneumonia in an external validation on an independent dual-center dataset. Our study revealed the high NPV of the tested AI solutions. However, specificity was highly variable, at 31–80%, and the PPV was considerably lower, at 19–25%. Sensitivity was high for one solution at 96%, while for the others it was 62–85%.

Several publications have claimed that deep-learning models can accurately detect COVID-19 pneumonia and thus differentiate it from other lung infections or normal lung parenchyma [10–13]. However, a systematic review came to an entirely different conclusion and stated that “currently, AI solutions are not of potential clinical use due to underlying methodological weaknesses or biases” [14].

At the time of the initial evaluation of our chest CT collection, all solutions were under development and not purchasable. To date, the training and validations of two of the evaluated algorithms have been published [18, 19]. Both algorithms were trained to detect chest CTs suspicious for COVID-19 pneumonia and achieved a sensitivity ≥ 0.90 and a specificity ≥ 0.83 in training.

Our study revealed a relatively high sensitivity for at least one solution, which was comparable to those published that focus on the detection of COVID-19 pneumonia with AI. However, in our study, specificity was considerably lower, with values of 31–80%, while previous studies have found specificities of 82–96% [4, 20].

Whereas the AUC was > 0.70 for three companies and 0.54 for the other company in the analysis of all CT studies with proven COVID-19 pneumonia, the AUC was lower in the subgroup analysis of studies with GGO: one of the AI solutions reached an AUC of 0.69, while the other three AI solutions performed even worse, with AUCs between 0.54 and 0.58. One tool, especially, generated many false-positive cases (86 false positives in 149 cases) in differentiating COVID-19 pneumonia from other conditions. PPV in our study ranged between 0.19 and 0.25. However, one solution yielded an acceptable sensitivity. In a scenario where not all patients are tested routinely, AI solutions have the potential to provide an alert tool for clinical routine, automatically identifying potential COVID-19 cases directly after image acquisition. Those patients might quickly be sent for a subsequent PCR test. However, our results indicate that further improvement of the algorithms is mandatory. Thus, large-scale training and evaluation studies should strengthen the performance of the solutions.

In our dataset, 10% of the cases had a proven SARS-CoV-2 infection. In previous studies, the external datasets for validation of deep-learning algorithms contained between 19 and 77% COVID-19 patients [10, 12]. Thus, the rate of COVID-19 pneumonia in our dataset was relatively low compared to previous studies. However, even this proportion may be relatively high, given the fact that, by chance, far fewer COVID-19–suspected lung changes will occur in clinical practice, especially when a large number of people have been vaccinated. A lower rate of COVID-19 patients within our dataset would result in an even lower specificity and a lower PPV. In this study, we additionally included the diagnoses of two board-certified radiologists and compared the results with those of the algorithms. We hypothesized that the performance of the algorithms might be strongly influenced by the nature of the disease: CT findings of COVID-19 pneumonia are nonspecific, and many different issues can be mimics. Thus, we decided to include radiologists in a direct comparison and found that they also had problems in classifying scans as “COVID-19 suspicious” or not. Interestingly, the sensitivity of radiologist 1 was only slightly below that of company 1 and above those of all the other algorithms, while the sensitivity of radiologist 2 lay within the range of sensitivities of the other algorithms. Regarding specificity and PPV, both radiologists were distinctly superior to the algorithms. However, a high sensitivity is especially required to avoid missing any patients with COVID-19 pneumonia. Company 1 had the overall highest sensitivity, with an acceptable value of 0.96. Thus, AI tools have the potential to support radiologists as an alert function to detect chest CT scans suspicious for COVID-19 pneumonia during clinical routine. Our study results indicate the high specificity of radiologists, who could then further triage the patients using clinical information, which enhances the certainty of image-based decision making tremendously [21]. However, our results indicate that further improvement of the algorithms is mandatory prior to routine clinical use.

In some studies, the algorithms had to differentiate between COVID-19 pneumonia and bacterial or viral pneumonias, whereas Harmon et al. used CT studies of any clinical indication or staging CTs. The high variability in the specificity of the AI solutions in differentiating COVID-19 pneumonia from other conditions is due to the broad spectrum of other pulmonary conditions that mimic those of COVID-19, such as atypical bacterial pneumonias, *Pneumocystis jirovecii* pneumonia, pulmonary edema, and hypersensitivity pneumonia [22]. Findings on CT are nonspecific and can overlap even with non-infectious diseases [23]. Our dataset is focused on real clinical data and not on specific diseases, except for the 50 COVID-19–positive patients.

Interrater reliability between the four AI solutions was very low. Only between one pair of AI solutions could a minimal agreement be found in the kappa statistic, whereas no agreement was present among all other cases. This fact is in line with the highly variable specificity and low positive predictive value in diagnosing COVID-19 pneumonia with CT in general, with or without AI [5]. Neri et al. state that “CT with artificial intelligence for screening or as first-line test to diagnose COVID-19” is not recommended [24]. Of course, nasal swab and PCR testing remain the mainstay of public screening, as CT imaging would not be justifiable. However, as mentioned above, CT-supporting AI solutions might function as an alert tool in daily clinical routine, especially in the analysis of CTs performed for another reason. However, our results indicate that further improvement of the algorithms is mandatory, as three solutions reached sensitivities below 90% despite being specifically trained for the identification of chest CT scans suspicious for COVID-19 pneumonia.

In this study, we sent only the raw DICOM files to the companies. Thus, all processing was in their hands and we received only the binary classification of our dataset. Consequently, we did not have an insight into the processing and the visualization of the results and the software’s possible usability and ease of integration into the routine workflow. Additionally, as all products were under development at the time of the image evaluation, we had no information regarding the prices and support services of the finalized products. Therefore, a more detailed comparison of the algorithms with regard to pricing, implementation into the routine workflow, support, etc. was not performed. Once an algorithm is commercially available, we suggest using the ECLAIR checklist to determine the optimal product [25].

Our study has limitations. First, we defined the composition of the dataset with regard to the proportion of CT studies with COVID-19 pneumonia versus other conditions at 10% versus 90%. As mentioned above, this proportion may be relatively high. However, a lower rate of COVID-19 patients would result in an even lower PPV. Second, we aimed to contact as many companies as possible, but other commercial solutions exist. With four AI solutions from European and Asian companies, this work offers a good insight into the market. Third, we sent the companies the raw DICOM files. Thus, we had no information about the segmentation accuracies, volume calculations, or probability scores of the algorithms. As an additional limitation, it is not possible to openly publish the names of the companies in relation to their results, due to data safety reasons and the lack of an agreement. Fourth, one company was able to analyze only CT studies without i.v. contrast. Thus, the number of cases in their dataset is considerably reduced, to 176. The interpretation of the results and comparability with the other algorithms, which analyzed a larger number of cases, are therefore limited. Fifth, our radiologists’ diagnoses were made without any knowledge of the patients’ symptoms or clinical histories. Clearly, this created an artificial reading situation but was essential to allow for comparison with the algorithms. Clinical information strengthens the differentiation between COVID-19 pneumonia and other lung pathologies [21].

In summary, this study highlights the relatively low and variable specificity and low PPV of commercial AI solutions in detecting COVID-19 pneumonia in chest CT. However, one solution yielded acceptable values for sensitivity. Thus, commercial AI solutions currently under development could potentially be integrated as alert tools for the detection of patients with lung changes suspicious for COVID-19 pneumonia in clinical routine workflow if further improvements are made. In general, AI has the potential to support radiologists in their daily practice. However, randomized trials are needed to assess AI’s true benefit and to critically consider any potential harms, since a large number of patients with false-positive findings would be diagnosed if AI were used [26]. Those trials should be performed prospectively and include a clinical validation to measure the impact of AI on treatment decisions.

## Supplementary Information

Below is the link to the electronic supplementary material.

Supplementary file1 (DOCX 51 KB)

## Abbreviations

COVID-19: Coronavirus disease 2019
GGO: Ground-glass opacity
RT-PCR: Real-time polymerase chain reaction
SARS-CoV-2: Severe acute respiratory syndrome coronavirus 2

## Declarations

### Guarantor

The scientific guarantor of this publication is PD Dr. med. Roman Kloeckner.

### Statistics and biometry

One of the authors has significant statistical expertise.

### Methodology

• retrospective.

• diagnostic or prognostic study.

• multicenter study.
