# Supplementary material for: Commercial AI solutions in detecting COVID-19 pneumonia in chest CT: not yet ready for clinical implementation?
Source: Eur Radiol. 2021 Dec 23;32(5):3152–60. doi: 10.1007/s00330-021-08409-4 (PMC8700707; doi:10.1007/s00330-021-08409-4)
Supplement: Supplementary file 1 — Supplementary file1 (DOCX 51 KB) [file 330_2021_8409_MOESM1_ESM.docx]

**Electronic Supplementary Material (ESM)**

**Supplementary Table 1.** Morphologic and clinical characteristics of the GGO subset

|  | | **Whole dataset (n = 150)** | **Proven SARS-CoV-2 infection (n = 48)** | **Other lung conditions (2018) (n = 102)** |
| --- | --- | --- | --- | --- |
| **Extensiveness, n (%)** | **Mild** | 52 (34.7) | 14 (29.2) | 38 (37.2) |
|  | **Moderate** | 51 (34.0) | 20 (41.6) | 31 (30.4) |
|  | **Marked** | 47 (31.3) | 14 (29.2) | 33 (32.4) |
| **Form, n (%)** | **Rounded** | 21 (14.0) | 6 (12.5) | 15 (14.7) |
|  | **Oval** | 36 (24.0) | 10 (20.8) | 26 (25.5) |
|  | **Lobulated** | 38 (25.3) | 16 (33.3) | 22 (21.6) |
|  | **Polygonal** | 55 (36.7) | 16 (33.3) | 39 (38.2) |
| **Predominant distribution horizontal, n (%)** | **Peripheral** | 74 (49.3) | 23 (47.9) | 51 (50.0) |
|  | **Perihilar** | 13 (8.7) | 3 (6.3) | 10 (9.8) |
|  | **Mixed** | 63 (42.0) | 22 (45.8) | 41 (40.2) |
| **Predominant distribution vertical, n (%)** | **Upper lung** | 35 (23.3) | 8 (16.7) | 27 (26.5) |
|  | **Lower lung** | 27 (18.0) | 7 (14.6) | 20 (19.6) |
|  | **Both** | 74 (49.3) | 29 (19.3) | 45 (44.1) |
|  | **In-between** | 14 (9.3) | 4 (8.3) | 10 (9.8) |
| **Lobar involvement,  n (%) *** | **Right upper lobe** | 113 (75.3) | 40 (83.3) | 73 (71.6) |
|  | **Right middle lobe** | 77 (51.3) | 30 (62.5) | 47 (46.1) |
|  | **Right lower lobe** | 87 (58.0) | 30 (62.5) | 57 (55.9) |
|  | **Left upper lobe** | 98 (65.3) | 36 (75.0) | 62 (60.8) |
|  | **Left lower lobe** | 96 (64.0) | 40 (83.3) | 56 (54.9) |
| **Density, n (%)** | **Pure** | 91 (60.7) | 28 (58.4) | 63 (61.8) |
|  | **Minor solid areas (<25%)** | 48 (32.0) | 16 (33.3) | 32 (31.4) |
|  | **Major solid areas (>25%)** | 11 (7.3) | 4 (8.3) | 7 (6.8) |
| **Causes, n (%)** | **COVID-19** | 48 (32.0) | 48 (100.0) | 0 |
|  | **Other viral infection** | 10 (6.7) | 0 | 10 (9.8) |
|  | **Non-viral infection** | 39 (26.0) | 0 | 39 (38.3) |
|  | **Mixed viral and non-viral infection** | 4 (2.7) | 0 | 4 (3.9) |
|  | **No pathogen detectable during hospitalization** | 22 (14.7) | 0 | 22 (21.6) |
|  | **Chronic lung diseases** | 14 (9.3) | 0 | 14 (13.7) |
|  | **Unspecific GGO found during staging, emergency diagnostics or treatment planning** | 13 (8.7) | 0 | 13 (12.7) |

*More than 100% possible as more than one lobe could be involved.

**Supplementary Table 2:** Reasons for imaging in the patients without PCR-proven SARS-CoV-2 infection

| **Reasons for imaging, n (%)** | **Other lung conditions (n = 450)** |
| --- | --- |
| Suspected pneumonia | 107 (23.8) |
| Viral pneumonia | 13 (2.9) |
| Non-viral pneumonia | 54 (12.0) |
| Bacterial | 42 (9.3) |
| Fungal | 13 (2.9) |
| Viral and non-viral pneumonia combined | 4 (0.9) |
| No pathogen for pneumonia detectable during the hospitalization | 35 (7.8) |
| Chronic lung disease | 22 (4.9) |
| Interstitial lung disease | 7 (1.6) |
| Chronic obstructive pulmonary disease | 2 (0.4) |
| Therapy-related pneumonitis | 10 (2.2) |
| Metabolic or autoimmune disease | 3 (0.7) |
| Involvement of the intestinal tissue | 26 (5.8) |
| Tumor staging | 294 (65.3) |
| Search for inflammatory foci during systemic inflammation | 15 (3.3) |
| Emergency diagnostics | 6 (1.3) |
| Treatment planning | 6 (1.3) |

**Supplementary Table 3.** Overview of the algorithms evaluated in this study

| **Company** | **Version used for the “COVID challenge”** | **Training goal** | **Training and validation set composition and initial results** | **Commercially available** | **Product certification** |
| --- | --- | --- | --- | --- | --- |
| Infervision, | InferRead CT Pneumonia version 1 | COVID-19 detection | As published^a^ | Yes (InferRead CT Pneumonia version 1) | CE-certification (class IIa) |
| Icometrix, | Icolung 0.6.0 | COVID-19 detection^b^ | Not published yet, no public information | Yes (Icolung 0.6.0) | CE-certification (FDA pending) |
| Siemens | Pneumonia Assessment V 2.0 | COVID-19 detection | As published^c^  10.1007/s00330-021-07937-3 | Yes (CT Pulmonary Density)^d^ | CE and FDA certification for CT Pulmonary Density^d^ |
| Contextflow, | Contexflow DEMO Lung CT 1.1.8 | COVID-19 detection | See below^e^ | Yes (contextflow SEARCH lung CT 1.2) | CE-certification for the pattern recognition modul contextflow SEARCH Lung CT 1.2 (no specific COVID-19 classification) |

^a^Wang M, Xia C, Huang L, et al. (2020) Deep learning-based triage and analysis of lesion burden for COVID-19: a retrospective study with external validation. Lancet Digit Health 2(10):e506–e515.

^b^Only in unenhanced CT scans.

^c^Mortani Barbosa EJ Jr, Georgescu B, Chaganti S, et al. (2021) Machine learning automatically detects COVID-19 using chest CTs in a large multicenter cohort. Eur Radiol 1:1–11.

^d^Segmentation of the lung and the COVID-19 affected areas, not for the COVID-19 probability estimation used for this study.

^e^According to contexflow, the COVID-19 classification model was trained on an internal dataset with 91 positive and 2700 negative patients and validated on 53 positive and 1424 negative patients. The trained model yielded an AUC of 0.89, a sensitivity of 0.75, a specificity of 0.87, and was used to predict the labels for the challenge.

**Fig 1.** STARD

|  | **Section & Topic** | **No** | **Item** | **Reported on page #** |
| --- | --- | --- | --- | --- |
|  |  |  |  |  |
|  | **TITLE OR ABSTRACT** |  |  |  |
|  |  | **1** | Identification as a study of diagnostic accuracy using at least one measure of accuracy  (such as sensitivity, specificity, predictive values, or AUC) | 1 |
|  | **ABSTRACT** |  |  |  |
|  |  | **2** | Structured summary of study design, methods, results, and conclusions  (for specific guidance, see STARD for Abstracts) | 1 |
|  | **INTRODUCTION** |  |  |  |
|  |  | **3** | Scientific and clinical background, including the intended use and clinical role of the index test | 4 |
|  |  | **4** | Study objectives and hypotheses | 5 |
|  | **METHODS** |  |  |  |
|  | *Study design* | **5** | Whether data collection was planned before the index test and reference standard  were performed (prospective study) or after (retrospective study) | 6 |
|  | *Participants* | **6** | Eligibility criteria | 6 |
|  |  | **7** | On what basis potentially eligible participants were identified  (such as symptoms, results from previous tests, inclusion in registry) | 6 |
|  |  | **8** | Where and when potentially eligible participants were identified (setting, location and dates) | 6 |
|  |  | **9** | Whether participants formed a consecutive, random or convenience series | 6 |
|  | *Test methods* | **10a** | Index test, in sufficient detail to allow replication | 6 |
|  |  | **10b** | Reference standard, in sufficient detail to allow replication | 6 |
|  |  | **11** | Rationale for choosing the reference standard (if alternatives exist) | 4,8 |
|  |  | **12a** | Definition of and rationale for test positivity cut-offs or result categories  of the index test, distinguishing pre-specified from exploratory | 6 |
|  |  | **12b** | Definition of and rationale for test positivity cut-offs or result categories  of the reference standard, distinguishing pre-specified from exploratory | 6 |
|  |  | **13a** | Whether clinical information and reference standard results were available  to the performers/readers of the index test | 6 |
|  |  | **13b** | Whether clinical information and index test results were available  to the assessors of the reference standard | 6 |
|  | *Analysis* | **14** | Methods for estimating or comparing measures of diagnostic accuracy | 7 |
|  |  | **15** | How indeterminate index test or reference standard results were handled | n/a |
|  |  | **16** | How missing data on the index test and reference standard were handled | n/a |
|  |  | **17** | Any analyses of variability in diagnostic accuracy, distinguishing pre-specified from exploratory | 9 |
|  |  | **18** | Intended sample size and how it was determined | 6, 10 |
|  | **RESULTS** |  |  |  |
|  | *Participants* | **19** | Flow of participants, using a diagram | n/a |
|  |  | **20** | Baseline demographic and clinical characteristics of participants | 6 |
|  |  | **21a** | Distribution of severity of disease in those with the target condition | 6 |
|  |  | **21b** | Distribution of alternative diagnoses in those without the target condition | 6 |
|  |  | **22** | Time interval and any clinical interventions between index test and reference standard | n/a |
|  | *Test results* | **23** | Cross tabulation of the index test results (or their distribution)  by the results of the reference standard | 9 |
|  |  | **24** | Estimates of diagnostic accuracy and their precision (such as 95% confidence intervals) | 9 |
|  |  | **25** | Any adverse events from performing the index test or the reference standard | n/a |
|  | **DISCUSSION** |  |  |  |
|  |  | **26** | Study limitations, including sources of potential bias, statistical uncertainty, and generalisability | 11 |
|  |  | **27** | Implications for practice, including the intended use and clinical role of the index test | 10,11,12 |
|  | **OTHER INFORMATION** |  |  |  |
|  |  | **28** | Registration number and name of registry | n/a |
|  |  | **29** | Where the full study protocol can be accessed | n/a |
|  |  | **30** | Sources of funding and other support; role of funders | 12 |
|  |  |  |  |  |
|  |  |  |  |  |

**
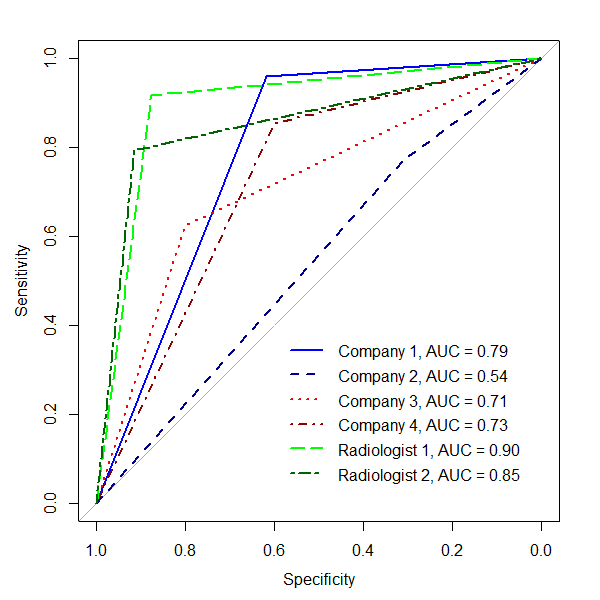
**

**Fig 2.** AUC for CO-RADS ≥3; company 1 (blue), company 2 (dark blue), company 3 (red), and company 4 (dark red).

**
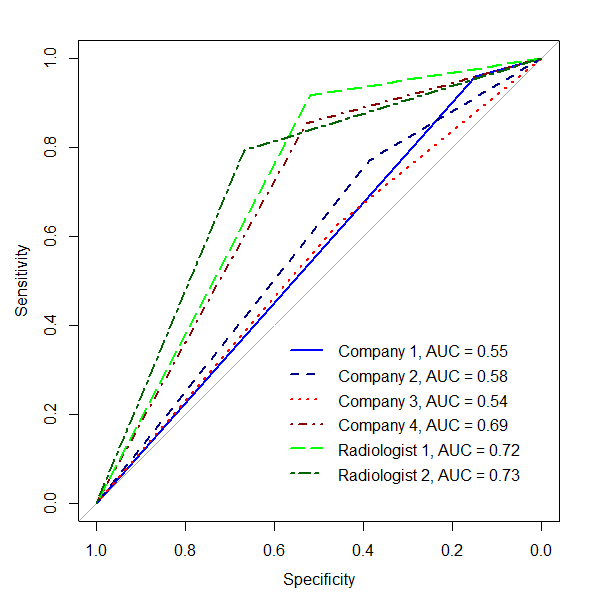
**

**Fig 3.** AUC for CO-RADS ≥3 in CT studies with GGO; company 1 (blue), company 2 (dark blue), company 3 (red), and company 4 (dark red).
